# Unveiling the enigma: Investigating the controversy surrounding mitochondrial DNA copy number and gastric cancer using Mendelian randomization analysis

**DOI:** 10.1097/MD.0000000000043916

**Published:** 2025-08-15

**Authors:** Jie Zhou, Taohua Shangguan, Yixin Xu, Chao Chen, Kun Wang

**Affiliations:** a Department of Gastrointestinal Surgery, The Wujin Hospital Affiliated with Jiangsu University, Changzhou, China; b Department of Gastrointestinal Surgery, The Wujin Clinical College of Xuzhou Medical University, Changzhou, China.

**Keywords:** causal relationship, genetically predicted, GWAS, single nucleotide polymorphism

## Abstract

Gastric cancer (GC), as one of the most prevalent malignant tumors, significantly impacts individuals’ health. Many studies have examined the relationship between mitochondrial DNA (mtDNA) copy number and GC. However, conclusions remain inconclusive, with conflicting findings. The genome-wide association study summary statistics for mtDNA copy number were obtained from 2 sources: one from a robust cohort of 465,809 White individuals provided by the Cohorts for Heart and Aging Research in Genomic Epidemiology consortium and the UK Biobank; and the other from a dataset comprising 395,718 UK Biobank participants. In addition, a total of 5 sets of genome-wide association study summary statistics for GC were obtained through datasets from Finland, the European Bioinformatics Institute, and the Integrative Epidemiology Unit at the University of Bristol, encompassing a total of 937,663 participants. Furthermore, we undertook a 2-sample bidirectional Mendelian randomization analysis to explore the association between mtDNA copy number and GC. The Inverse variance weighted (IVW) method was primarily utilized, complemented by 4 other validation methods. Based on our comprehensive investigation, no discernible causal relationship was found between mtDNA copy number and GC in both the training and validation cohorts (IVW, *P >*.05). Furthermore, in the reverse Mendelian randomization analysis, no association was observed between GC and mtDNA copy number either (IVW, *P >*.05). Additionally, all analyses showed no evidence of horizontal pleiotropy or heterogeneity. The findings of this study provide evidence that there is no causal relationship between mtDNA copy number and GC.

## 1. Introduction

Worldwide, gastric cancer (GC) remains one of the most common malignancies and a leading cause of mortality. According to the latest estimates released by GLOBOCAN, the annual incidence of GC worldwide reached 1089,000 cases, ranking fifth among all malignant tumors.^[1]^ While incidence rates are declining in many countries, the aging demographic profile is anticipated to drive a future increase in GC cases.^[2]^ The challenge of timely detection of this insidious cancer for effective therapeutic interventions persists. In recent years, the exploration of GC risk factors and the identification of reliable screening markers continue to represent critical areas of research.^[3]^

Mitochondria play a crucial role in the fundamental functions of eukaryotic cells, such as energy production, ROS regulation, autophagy, senescence, and involvement in cell signaling pathways.^[4]^ Mitochondrial DNA (mtDNA), the unique genome of mitochondria, encodes 2 ribosomal RNAs, 22 transfer RNAs, and 13 polypeptides essential for the respiratory chain.^[5]^ The mitochondrial DNA copy number reflects the ratio of mitochondrial to nuclear DNA copies, serving as a surrogate marker for mitochondrial quantity and dysfunction, indirectly reflecting damage to mtDNA.^[6,7]^ Additionally, the mtDNA copy number can vary under different physiological and stress conditions (such as ROS/oxidative stress) and may be associated with pathological processes like cancer, diabetes, and Huntington disease.^[7,8]^

Currently, there is a significant controversy regarding the relationship between mtDNA copy number and GC. On one hand, Zhu et al conducted a case-control study and measured the relative mtDNA copy number in peripheral blood leukocytes using real-time quantitative PCR. Their findings suggested a potential positive involvement of mtDNA in the development of GC.^[9]^ Similarly, Fernandes et al also observed that the peripheral blood mtDNA copy number in GC patients was significantly higher than in the noncancer control group.^[10]^ Additionally, Alikhani et al reported similar results in their study.^[11]^ On the other hand, Jiang et al analyzed the mtDNA copy numbers in the peripheral blood of high-altitude residents and found no significant difference in mtDNA copy numbers between GC patients and healthy individuals.^[12]^ The study by Liao et al also confirmed that there is no association between leukocyte mtDNA copy number and the risk of developing GC.^[13]^ Similarly, Lee et al reported comparable findings in their study.^[14]^ Even more surprisingly, Sun et al discovered that individuals with low mtDNA copy number had a significantly higher risk of developing GC.^[15]^ Given this, the causal association between mtDNA copy number and GC has not yet been established and requires further research.

Mendelian randomization (MR) is a potential causal inference method that utilizes genetic variations as instrumental variables (IVs) to infer the impact of exposure factors on outcomes from observational data.^[16]^ MR can mitigate the effects of unmeasured confounders or biases while leveraging Mendelian inheritance patterns to avoid reverse causation.^[16]^ In this study, we collected multiple recently published genome-wide association study (GWAS) summary statistics on GC and mtDNA copy number. Through 2-sample MR analysis, our primary aim is to elucidate the causal relationship between mtDNA copy number and GC, uncovering the pathogenesis of GC, and exploring potential therapeutic targets.

## 2. Methods

### 2.1. Study design

The data utilized in our analysis were sourced from publicly available repositories and had been approved by the institutional review committees of the respective studies. Therefore, no ethical committee review was deemed necessary for this study.

In this study, we explored the causal relationship between mtDNA copy number and GC through 2-sample bidirectional MR analyses, with single nucleotide polymorphisms (SNPs) serving as IVs.^[17]^ Three assumptions must be satisfied for MR analysis: the SNPs are linked to the exposure; the SNPs are independent of confounders in the exposure-outcome relationship; and the SNPs influence the outcome solely through the exposure.^[18]^

### 2.2. GWAS summary data sources

#### 2.2.1. mtDNA copy number

The GWAS summary statistics for mtDNA copy number were obtained from a robust cohort of 465,809 White individuals sourced from the esteemed Cohorts for Heart and Aging Research in Genomic Epidemiology consortium and the UK Biobank,^[19]^ serving as the primary training cohort, as detailed by Longchamps et al in their 2022 publication. To fortify the credibility of our findings, we introduced a validation group comprising 395,718 UK Biobank participants with diverse ancestries, predominantly of European descent.^[20]^ In addition, the GWAS was adjusted for age, age^2^, sex, chip type, 20 genetic principal components, and blood cell counts (including white blood cell, platelet, and neutrophil counts). This represents a more comprehensive genetic assessment of mtDNA copy number compared to previous studies.^[19,21]^

#### 2.2.2. Gastric cancer

In this study, we incorporated a total of 5 sets of GWAS summary statistics for GC as outcome data for analysis. Among these, we gathered 3 cohorts from Finland (DF10, Public release: December 18, 2023), which encompassed Adenocarcinoma and papillary adenocarcinoma of the stomach (AdenoPapAdenoCA) (n = 792, control = 314,193), Neuroendocrine tumor and carcinoma of the stomach (NETC) (n = 118, control = 314,193), and Malignant neoplasm of the stomach (MNOS) (n = 1423, control = 314,193). While there may be some overlap in these datasets, they can indirectly reflect the association between mtDNA copy number and different pathological subtypes of GC. Furthermore, this study obtained 2 large-scale GWAS datasets on GC from the European Bioinformatics Institute (https://www.ebi.ac.uk/gwas/) and the MRC Integrative Epidemiology Unit at the University of Bristol (https://gwas.mrcieu.ac.uk/). More specifically, the GC GWAS data were respectively analyzed by Sakaue et al (n = 7921, controls = 159,201)^[22]^ and Jiang et al (sample size = 456,348).^[23]^ The detailed information and sources of the aforementioned data can be found in Table 1.

**Table 1:** Details of the GWAS and datasets used in our analyses.

Phenotypes	Cases/controls	Consortium/Author	PubMed ID	Data download link
Mitochondrial DNA copy number	395,718	UK Biobank	35023831	https://www.ebi.ac.uk/gwas/
Mitochondrial DNA copy number in the replication analysis	465,809	CHARGE and UK Biobank	34859289	https://www.ebi.ac.uk/gwas/
Adenocarcinoma and papillary adenocarcinoma of stomach	792/314,193	FinnGen consortium	–	https://storage.googleapis.com/finngen-public-data-r10/summary_stats/finngen_R10_C3_STOMACH_ADENO_EXALLC.gz
Neuroendocrine tumor and carcinoma of stomach	118/314193	FinnGen consortium	–	https://storage.googleapis.com/finngen-public-data-r10/summary_stats/finngen_R10_C3_STOMACH_NEUROENDOCRINE_EXALLC.gz
Malignant neoplasm of stomach	1423/314,193	FinnGen consortium	–	https://storage.googleapis.com/finngen-public-data-r10/summary_stats/finngen_R10_C3_STOMACH_EXALLC.gz
Gastric cancer	7921/159,201	Saori Sakaue et al.	34594039	https://gwas.mrcieu.ac.uk/;ID: ebi-a-GCST90018629
Gastric cancer	456,348	Longda Jiang et al.	34737426	https://www.ebi.ac.uk/gwas/; ID: GCST90018849

GWAS = genome-wide association studies.

### 2.3. IVs selection and data harmonization

In our analysis, we included SNPs that showed genome-wide significance (*P < *5 × 10^−8^). These SNPs were then grouped based on linkage disequilibrium (using a window size of 10,000 kb and *r*^2^ < 0.001). Moreover, palindromic and ambiguous SNPs were excluded from the IVs utilized in the MR analysis.^[24]^ However, if selecting based on *P* *<* 5 × 10^−8^ results in a limited number of SNPs, we will further relax the selection criteria (e.g., using *P* *<* 1 × 10^−5^) for refinement. The F statistic was determined by assessing the variance elucidated by the SNPs for each exposure, computed as [(N—K—1)/K]/ [R2/ (1–R2)], where K signifies the count of genetic variants and N represents the sample size. To uphold the robustness and dependability of the findings, we omitted feeble IVs (F-statistics < 10) from the analysis.

### 2.4. Statistical analysis

For our meticulous and thorough analysis, we performed MR analysis utilizing R software (version 4.2.0, http://www.r-project.org) in tandem with the “Two-Sample MR” package (version 0.5.6).^[25]^ Additionally, we employed the online tool PhenoScanner to evaluate all established phenotypes associated with the genetic instruments under consideration in our analyses (http://www.phenoscanner.medschl.cam.ac.uk/).

### 2.5. Primary analysis

We conducted a 2-sample MR analysis to investigate whether genetically predicted mtDNA copy number has a causal relationship with GC. The Inverse Variance Weighted (IVW) method, as the most crucial and commonly used approach, employs meta-analysis to amalgamate the Wald ratios of causal effects for each SNP.^[26,27]^ In addition to IVW, we utilized supplementary methods including Weighted Median,^[28]^ MR-Egger,^[29]^ Simple mode and weighted mode approaches. A range of methodologies, each tailored to specific validity assumptions, were utilized to obtain MR estimates. IVW, for instance, operates under the assumption that all SNPs act as valid IVs, ensuring accurate estimations. The Weighted Median method showcases heightened precision, evident in its reduced standard deviation compared to MR-Egger analysis. Furthermore, the reliability of the results was further validated using data from a validation cohort.

### 2.6. Reverse MR analysis

In order to explore whether there is a reverse causal relationship between genetically predicted mtDNA copy number and GC, this study conducted a reverse MR analysis, treating GC as the exposure and mtDNA copy number as the outcome. Building upon the results from the IVW method, additional analysis methods were employed to validate the findings and further confirm their robustness through the utilization of data from a validation cohort.

### 2.7. Sensitivity analysis

Due to variations in experimental conditions, selected populations, and SNPs, heterogeneity may exist in 2-sample MR analyses, potentially leading to biased estimates of causal effects. Therefore, this study performed heterogeneity tests for the primary IVW and MR-Egger methods. Cochrane *Q* value was employed to evaluate the heterogeneity of the genetic instruments, where a *P*-value *>*.05 suggests no significant heterogeneity. Additionally, an essential assumption in MR analysis is that IVs affect the outcome solely through the exposure, highlighting the need to investigate potential horizontal pleiotropy between the exposure and outcome.^[30]^ In this study, the MR-Egger intercept method was utilized to evaluate the presence of pleiotropy. A *P*-value >.05 indicates a minimal or negligible probability of pleiotropy in the causal analysis, thereby permitting its exclusion. Finally, outliers in IVW analysis methods can be identified and adjusted using the MR-PRESSO test,^[31]^ while leave-one-out analysis was employed to determine the influence of a single SNP on the genetic causal relationship between the exposure and outcome.^[32]^

## 3. Results

### 3.1. Association of genetically predicted mtDNA copy number with GC in training cohort

After LD clumping, proxy SNP exploration, the Phenoscanner database mining and data harmonization, we selected eligible SNPs as IVs to fit 3 key assumptions. A total of 70 SNPs associated with mtDNA copy number were obtained for further analysis in the training cohord (Table S1, Supplemental Digital Content, https://links.lww.com/MD/P685).

To investigate the genetically predicted causal relationship between the mtDNA copy number and GC, we used the mtDNA copy number dataset from the training cohort as the exposure and datasets from 5 groups of GC datasets as the outcome for MR analysis.

**Figure 1.**
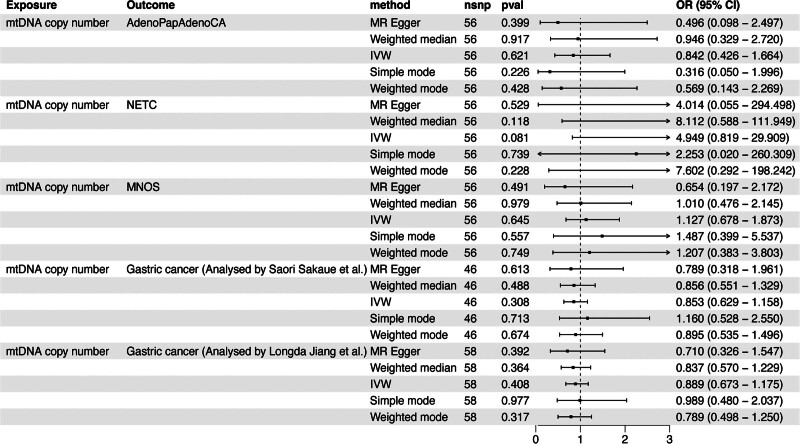
The MR analysis between mtDNA copy number and GC in the training cohort. AdenoPapAdenoCA = adenocarcinoma and papillary adenocarcinoma of the stomach, GC = gastric cancer, IVW, Inverse variance weighted, MNOS = malignant neoplasm of the stomach, MR = Mendelian randomization, mtDNA = mitochondrial DNA, NETC = neuroendocrine tumor and carcinoma of the stomach.

In the Finnish database, within the first dataset on GC, characterized by the phenotype of AdenoPapAdenoCA, a systematic MR analysis did not uncover any evidence of a genetically predicted causal relationship between mtDNA copy number and GC (IVW: odds ratio [OR], 0.842; 95% confidence interval [CI], 0.426–1.664; *P* *=* .621). The second dataset, characterized by the phenotype of NETC, was incorporated into the MR analysis, revealing no association between mtDNA copy number and GC (IVW: OR, 4.949; 95% CI, 0.819–29.909; *P =* .081). In the third dataset, characterized by MNOS as the outcome, no causal relationship with mtDNA copy number was similarly found (IVW: OR, 1.127; 95% CI, 0.678–1.873, *P* *=* .645) (Fig. 1).

Subsequently, we continued the analysis of 3 large-scale GWAS datasets on GC sourced from the European Bioinformatics Institute and the MRC Integrative Epidemiology Unit at the University of Bristol. Using GWAS data analyzed by Sakaue et al, no causal relationship between mtDNA copy number and GC was identified (IVW: OR, 0.853; 95% CI, 0.629–1.158; *P* *=* .308). Furthermore, in the data analyzed by Jiang et al, consistent negative results were obtained (IVW: OR, 0.889; 95% CI, 0.673–1.175; *P* *=* .408) (Fig. 1).

In the above-mentioned 5 MR analyses concerning mtDNA copy number and GC, various analytical methods, including Weighted Median, MR-Egger, Simple Mode, and Weighted Mode analyses, were incorporated. The results revealed that all findings aligned with the primary IVW method, failing to establish a significant association between the 2 factors (Table S2, Supplemental Digital Content, https://links.lww.com/MD/P685).

### 3.2. Reverse MR analysis in the training cohort

To delve deeper into the association between GC and mtDNA copy number, we conducted a reverse MR analysis, treating GC as the exposure and mtDNA copy number as the outcome. Given the limited number of SNPs obtained using a stringent threshold of *P <* 5 × 10^−8^, we chose a more inclusive criterion of *P <* 1 × 10^−5^ for screening in this study.

**Figure 2.**
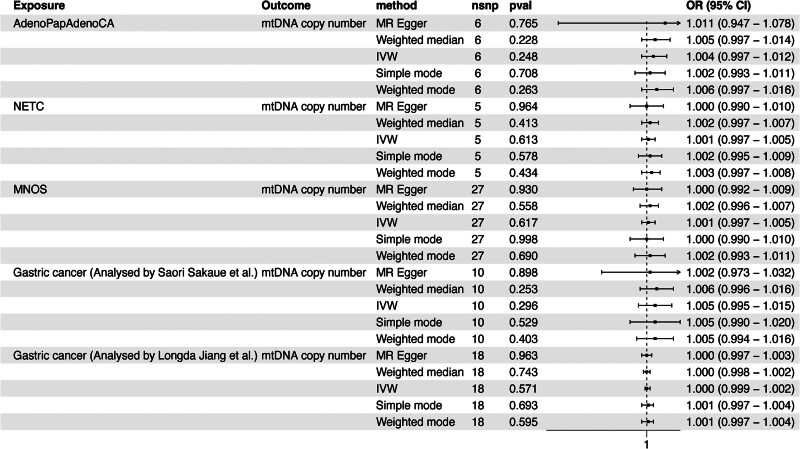
The reverse MR analysis between mtDNA copy number and GC in the training cohort. AdenoPapAdenoCA = adenocarcinoma and papillary adenocarcinoma of the stomach, GC = gastric cancer, IVW, Inverse variance weighted, MNOS = malignant neoplasm of the stomach, MR = Mendelian randomization, mtDNA = mitochondrial DNA, NETC = neuroendocrine tumor and carcinoma of the stomach.

In the Finnish database, with phenotypes including AdenoPapAdenoCA (IVW: OR, 1.004; 95% CI, 0.997–1.012; *P =* .248), NETC (IVW: OR, 1.001; 95% CI, 0.997–1.005; *P =* .613), and MNOS (IVW: OR, 1.001; 95% CI, 0.997–1.005; *P = *.617) as exposures, no reverse causal relationship was found between GC and mtDNA copy number (Fig. 2).

Additionally, we included GWAS datasets on GC separately analyzed by Sakaue et al (IVW: OR, 1.005; 95% CI, 0.995–1.015; *P =* .296) and Jiang et al (IVW: OR, 1.000; 95% CI, 0.999–1.002, *P =* .571; Outlier SNP removed: rs17242869) as exposures to explore their causal relationship with mtDNA copy number. Similar to the results from the Finnish dataset, none of these 3 datasets revealed a reverse causal relationship with mtDNA copy number (Fig. 2).

In all the reverse MR analyses mentioned above, we also employed a variety of analytical techniques, such as weighted median, MR-Egger, simple mode, and weighted mode methods. Consistently, all findings aligned with the primary IVW method, indicating no significant association between these 2 factors (Table S3, Supplemental Digital Content, https://links.lww.com/MD/P685).

### 3.3. Conducting bidirectional MR analysis in the validation cohort

We performed validation analysis using the GWAS summary statistics for mtDNA copy number on 395,718 UK Biobank participants, predominantly of European descent. After LD clumping, proxy SNP exploration, the Phenoscanner database mining and data harmonization, 68 SNPs associated with mtDNA copy number were selected for further analysis in the validation cohort (Table S4, Supplemental Digital Content, https://links.lww.com/MD/P685).

**Figure 3.**
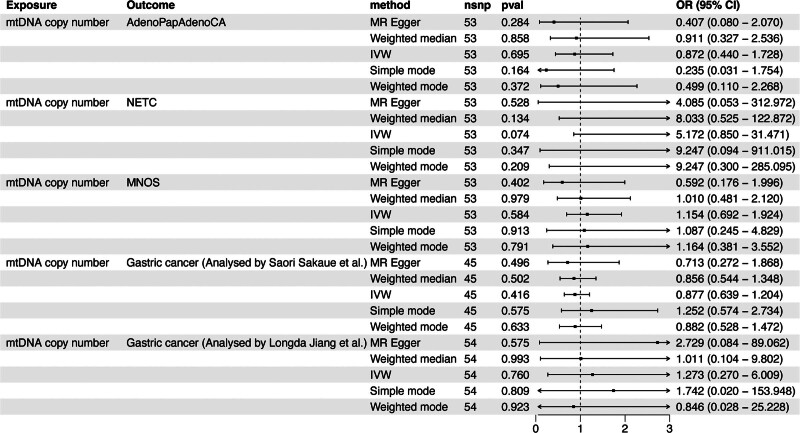
The MR analysis between mtDNA copy number and GC in the validation cohort. AdenoPapAdenoCA = adenocarcinoma and papillary adenocarcinoma of the stomach, GC = gastric cancer, IVW, Inverse variance weighted, MNOS = malignant neoplasm of the stomach, MR = Mendelian randomization, mtDNA = mitochondrial DNA, NETC = neuroendocrine tumor and carcinoma of the stomach.

In the Finnish database, with phenotypes including AdenoPapAdenoCA (IVW: OR, 0.872; 95% CI, 0.440–1.728; *P = *.695), NETC (IVW: OR, 5.172; 95% CI, 0.850–31.471; *P = *.074), and MNOS (IVW: OR, 1.154; 95% CI, 0.692–1.924; *P =* .584) as outcomes, no causal relationship was found between mtDNA copy number and GC. Additionally, similar results were found in GWAS datasets on GC separately analyzed by Sakaue et al (IVW: OR, 0.877; 95% CI, 0.639–1.204; *P* = .416) and Jiang et al (IVW: OR, 1.273; 95% CI, 0.270–6.009; *P* = .760) (Fig. 3) (Table S5, Supplemental Digital Content, https://links.lww.com/MD/P685).

**Figure 4.**
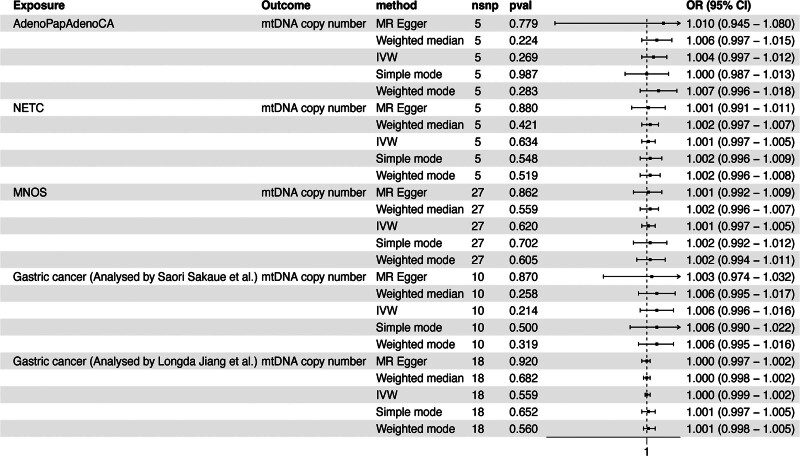
The reverse MR analysis between mtDNA copy number and GC in the validation cohort. AdenoPapAdenoCA = adenocarcinoma and papillary adenocarcinoma of the stomach, GC = gastric cancer, IVW, Inverse variance weighted, MNOS = malignant neoplasm of the stomach, MR = Mendelian randomization, mtDNA = mitochondrial DNA, NETC = neuroendocrine tumor and carcinoma of the stomach.

In the validation cohort, during the reverse MR analysis with phenotypes like adenocarcinoma and papillary adenocarcinoma of stomach (IVW: OR, 1.004; 95% CI, 0.997–1.012; *P* = .269), neuroendocrine tumor and carcinoma of stomach (IVW: OR, 1.001; 95% CI, 0.997–1.005; *P* = .634), and malignant neoplasm of stomach (IVW: OR, 1.001; 95% CI, 0.997–1.005; *P* = .620) as exposures, no causal link was established between GC and mtDNA copy number. Furthermore, similar results were observed in GWAS datasets on GC separately analyzed by Sakaue et al (IVW: OR, 1.006; 95% CI, 0.996–1.016; *P* = .214) and Jiang et al (IVW: OR, 1.000; 95% CI, 0.999–1.002; *P* = .559; Outlier SNP removed: rs17242869) (Fig. 4) (Table S6, Supplemental Digital Content, https://links.lww.com/MD/P685).

### 3.4. Sensitivity analysis

In the analyses mentioned above, we utilized the online tool PhenoScanner to evaluate each SNP, and the results revealed no association between these SNPs and the investigated outcomes. During the outlier test, we only identified an outlier in the reverse MR analysis when a GWAS dataset on GC separately analyzed by Longda Jiang et al was used as the exposure: rs17242869, which was subsequently removed from further analysis. Moreover, we employed a “leave-one-out” approach for sensitivity analysis to explore whether specific SNPs influenced the causal relationships. The results indicated that systematically excluding each SNP did not result in significant changes in the model’s effect estimates or qualitative conclusions (Fig. S1–4, Supplemental Digital Content, https://links.lww.com/MD/P684). Additionally, in all the analyses involving the training cohort, validation cohort, and all reverse MR analyses, no evidence of heterogeneity and horizontal pleiotropy was found (Table S7, Supplemental Digital Content, https://links.lww.com/MD/P685).

## 4. Discussion

Over the past decade, numerous observational studies have investigated the association between mtDNA copy number and GC. They have also explored the potential of peripheral blood mtDNA copy number as an early detection marker for GC. However, due to the influence of various confounding factors, studies in this field have produced inconsistent and even conflicting results.^[9–15]^ In this study, we have leveraged existing GWAS data to introduce an innovative approach. We have utilized MR analysis to establish a link between mtDNA copy number and GC. Through the application of 2-sample bidirectional MR analysis, our aim is to delve into the causal relationship between the 2 factors and identify potential therapeutic targets for GC.

Wu et al first described the changes in mtDNA copy numbers in GC.^[33]^ In comparison to corresponding noncancerous tissue, 50% of GC patients exhibited a relative decrease in mtDNA copy numbers, while 23% showed a relative increase. Additionally, the mtDNA copy number was associated with Borrmann type, indicating that the decrease in mtDNA may be involved in the phenotypic changes, tumor progression, and metastasis of GC. However, our study analyzed 5 sets of GWAS summary statistics for GC and 2 sets for mtDNA copy number. Through MR analysis, we did not find any causal relationship between mtDNA copy number and GC in both the training and validation cohort. Similarly, in the reverse MR analysis, negative results were obtained in both the training and validation cohort. Furthermore, tests for horizontal pleiotropy, heterogeneity analysis, and sensitivity analysis all met the 3 core assumptions of MR, further validating the reliability of our results. These findings are consistent with previous research results.^[12,13,15]^

The presence of numerous confounding factors often leads to inconsistent results. Similarly, according to Yu et al review of mitochondrial DNA copy numbers in different cancers, conflicting conclusions have also emerged in certain types of cancer.^[34]^ The observed increase in mitochondrial DNA may be attributed to the use of varying methods for estimating mitochondrial DNA content and DNA extraction. Therefore, to comprehend the significance of mitochondrial DNA alterations in cancer, standardized methods should be employed for calculating mitochondrial DNA content. In addition, factors such as the study population, sample size, statistical differences, and technical limitations are all reasons that can lead to biases in research results. Therefore, by using genetic variants as IVs, MR can mitigate the influence of unmeasured confounders or biases. This allows for the inference of the impact of mtDNA copy number on GC from observational data, leading to higher credibility of the conclusions.

To our knowledge, our study is the most comprehensive and largest MR analysis on the genetic causal effects of mtDNA copy number on the risk of GC. Our MR study boasts several strengths. Firstly, we employed a 2-sample MR design to mitigate the impact of confounding variables and reverse causation often encountered in traditional observational studies. Furthermore, we meticulously screened exposure-related IVs and manually excluded SNPs associated with potential confounders, thereby adhering closely to the fundamental assumptions of MR analysis. Lastly, our study conducted analyses across multiple datasets and validated our findings using a validation cohort, with results consistently supporting our conclusions.

Our study has several limitations. Firstly, since our genomic analysis was primarily conducted in individuals of European ancestry, and given the well-documented geographic variations in GC incidence and risk factors, the generalizability of our findings to other ethnic populations may be limited. Furthermore, the use of GWAS summary-level data inherently restricted our ability to perform more sophisticated ancestry heterogeneity controls, such as principal component analysis or population-specific stratification adjustments. Secondly, despite conducting various sensitivity analyses, we cannot completely rule out the potential impact of pleiotropies or heterogeneities. Moreover, the mtDNA GWAS data (e.g., UK Biobank) and partial GC GWAS datasets (e.g., Sakaue et al) may share overlapping participants, which could introduce bias in MR analysis. Although we performed sensitivity analyses using GC data from nonoverlapping cohorts (e.g., Finnish populations), residual confounding from undetected overlap cannot be entirely ruled out. Lastly, in the reverse MR analysis, constrained by a limited number of SNPs, we included SNPs with genome-wide significance (*P* < 1 × 10^−5^), which may have reduced the robustness of our conclusions.

## 5. Conclusion

In light of the substantial controversy surrounding the causal relationship between mtDNA copy number and GC, we conducted a meticulous analysis using MR on 2 GWAS datasets for mtDNA copy number and 5 GWAS datasets for GC. Our comprehensive investigation revealed no discernible causal relationship between peripheral blood mtDNA copy number and GC. Notably, the absence of positive findings in the reverse MR analysis further strengthens this conclusion.

## Author contributions

**Conceptualization:** Jie Zhou.

**Data curation:** Jie Zhou, Taohua Shangguan, Chao Chen, Kun Wang.

**Funding acquisition:** Yixin Xu.

**Methodology:** Jie Zhou, Kun Wang.

**Software:** Yixin Xu.

**Supervision:** Taohua Shangguan.

**Writing – original draft:** Jie Zhou.

**Writing – review & editing:** Taohua Shangguan.

## Supplementary Material


