# Supplementary material for: Unveiling the enigma: Investigating the controversy surrounding mitochondrial DNA copy number and gastric cancer using Mendelian randomization analysis
Source: Medicine (Baltimore). 2025 Aug 15;104(33):e43916. doi: 10.1097/MD.0000000000043916 (PMC12366980; doi:10.1097/MD.0000000000043916)

**Supplementary Figure S1:** MR leave-one-out sensitivity analysis for mDNA copy number on gastric cancer in the training cohort.


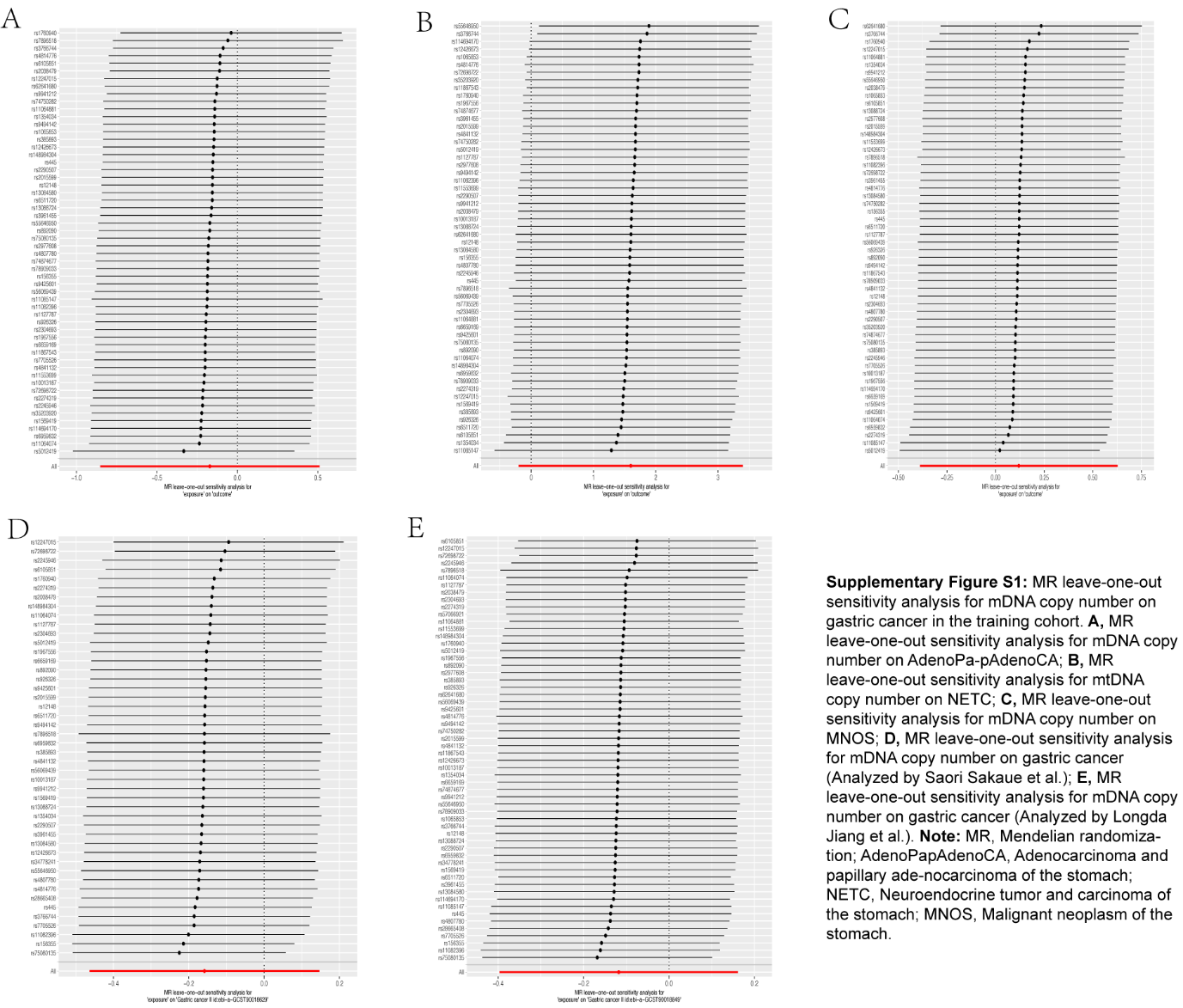


**Supplementary Figure S2:** MR leave-one-out sensitivity analysis for gastric cancer on mDNA copy number in the training cohort.


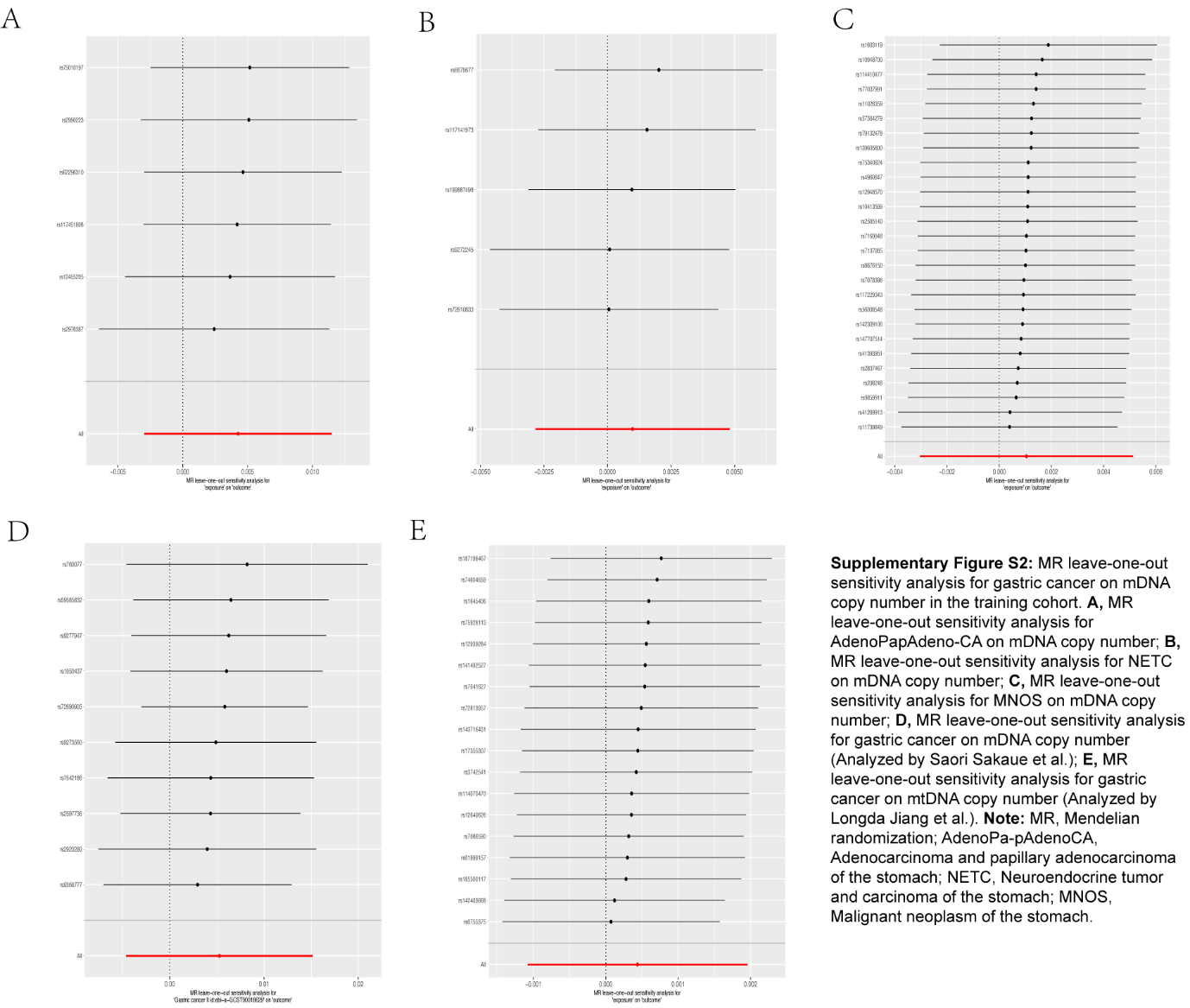


**Supplementary Figure S3:** MR leave-one-out sensitivity analysis for mDNA copy number on gastric cancer in the validation cohort.

**
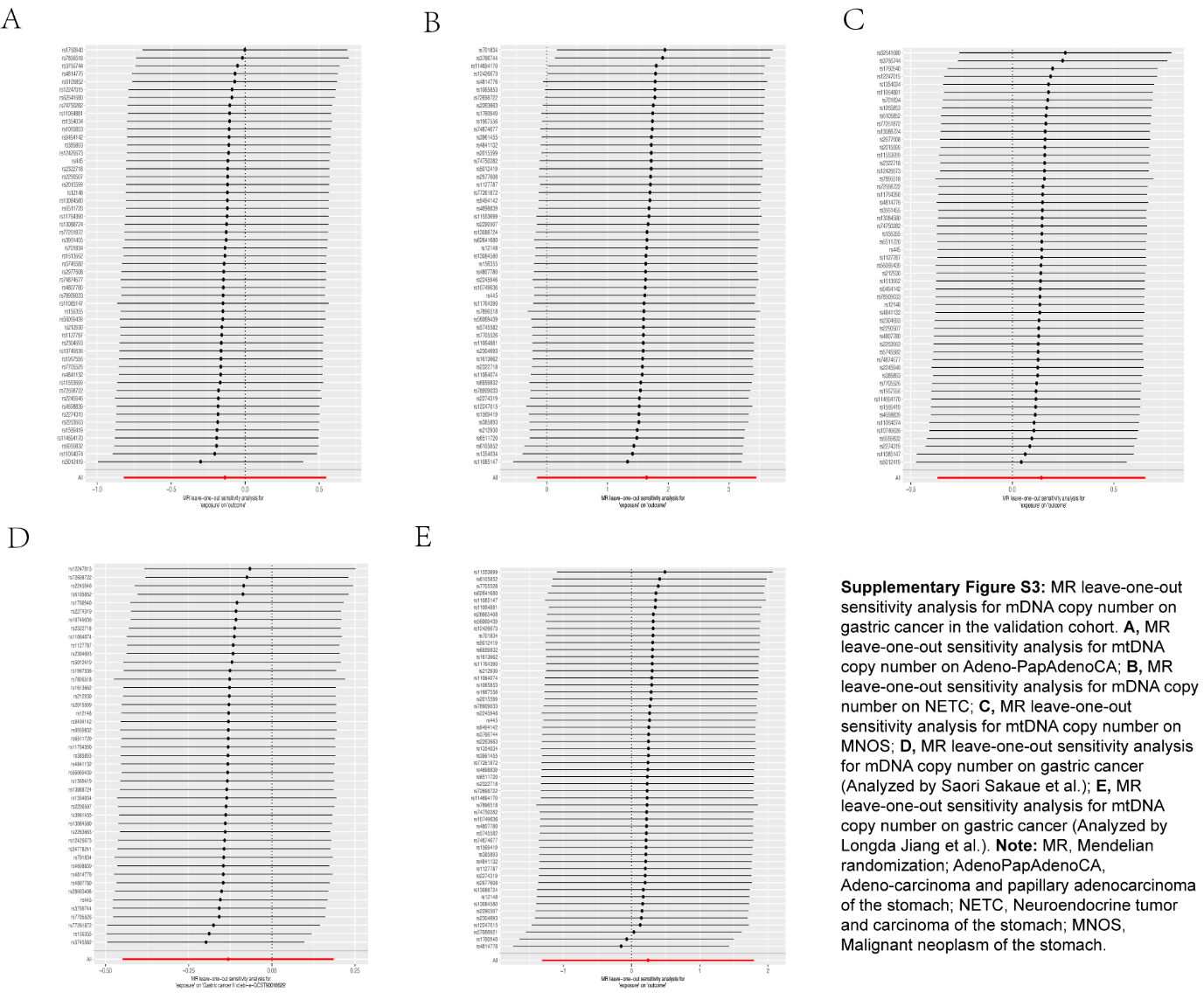
**

**Supplementary Figure S4:** MR leave-one-out sensitivity analysis for gastric cancer on mtDNA copy number in the validation cohort.


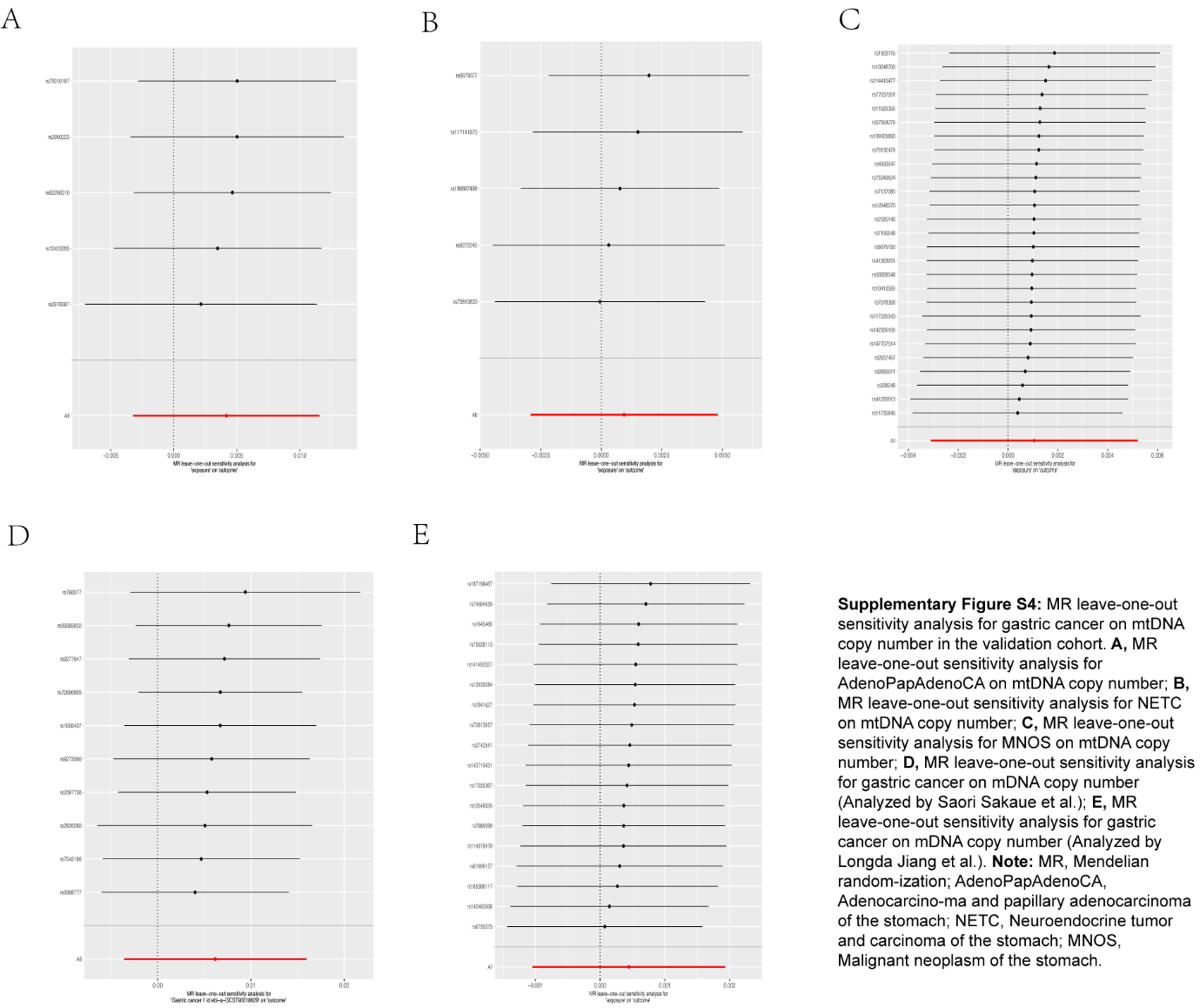

Supplement: Supplementary file 2 [file medi-104-e43916-s002.docx]
